# Sex differences in placenta-derived markers and later autistic traits in children

**DOI:** 10.1038/s41398-023-02552-w

**Published:** 2023-07-13

**Authors:** A. Tsompanidis, L. Blanken, Z. A. Broere-Brown, B. B. van Rijn, S. Baron-Cohen, H. Tiemeier

**Affiliations:** 1 Autism Research Centre, Department of Psychiatry, University of Cambridge, Cambridge, UK; 2 The Generation R Study Group, Erasmus MC, University Medical Centre, Rotterdam, The Netherlands; 3 Department of Child and Adolescent Psychiatry/Psychology, Erasmus MC, University Medical Centre, Rotterdam, The Netherlands; 4 Department of Obstetrics and Gynaecology, Erasmus MC, University Medical Centre, Rotterdam, The Netherlands; 5 Department of Social and Behavioral Sciences, Harvard T.H. Chan School of Public Health, Harvard University, Boston, USA

**Keywords:** Predictive markers, Physiology

## Abstract

Autism is more prevalent in males and males on average score higher on measures of autistic traits. Placental function is affected significantly by the sex of the fetus. It is unclear if sex differences in placental function are associated with sex differences in the occurrence of autistic traits postnatally. To assess this, concentrations of angiogenesis-related markers, placental growth factor (PlGF) and soluble fms-like tyrosine kinase (sFlt-1) were assessed in maternal plasma of expectant women in the late 1^st^ (mean= 13.5 [SD = 2.0] weeks gestation) and 2^nd^ trimesters (mean=20.6 [SD = 1.2] weeks gestation), as part of the Generation R Study, Rotterdam, the Netherlands. Subsequent assessment of autistic traits in the offspring at age 6 was performed with the 18-item version of the Social Responsiveness Scale (SRS). Associations of placental protein concentrations with autistic traits were tested in sex-stratified and cohort-wide regression models. Cases with pregnancy complications or a later autism diagnosis (*n* = 64) were also assessed for differences in placenta-derived markers. sFlt-1 levels were significantly lower in males in both trimesters but showed no association with autistic traits. PlGF was significantly lower in male pregnancies in the 1^st^ trimester, and significantly higher in the 2^nd^ trimester, compared to female pregnancies. Higher PlGF levels in the 2^nd^ trimester and the rate of PlGF increase were both associated with the occurrence of higher autistic traits (PlGF-2^nd^: *n* = 3469,*b* = 0.24 [SE = 0.11], *p* = 0.03) in both unadjusted and adjusted linear regression models that controlled for age, sex, placental weight and maternal characteristics. Mediation analyses showed that higher autistic traits in males compared to females were partly explained by higher PlGF or a faster rate of PlGF increase in the second trimester (PlGF-2^nd^: *n* = 3469, ACME: *b* = 0.005, [SE = 0.002], *p* = 0.004). In conclusion, higher PlGF levels in the 2^nd^ trimester and a higher rate of PlGF increase are associated with both being male, and with a higher number of autistic traits in the general population.

## Introduction

It has been hypothesised that sex differences in prenatal physiology may be contributing to sex differences in neurodevelopmental outcomes and autism [1]. Male fetuses are on average exposed to higher levels of sex steroid hormones, such as testosterone, during a period in the late first and second trimester of gestation, between 8 and 17 weeks gestational age, following the activation of the fetal testes [2]. One retrospective study found that several sex steroid hormones, and estrogens in particular, were higher in the amniotic fluid of individuals who were later diagnosed as autistic, compared to pregnancies where the individual developed neurotypically [3, 4]. Amniotic testosterone levels have also been found to predict autistic traits, in some but not all cohorts [5–7]. In terms of the maternal circulation, estrogen levels have been associated with the child’s later level of autistic traits as well as a clinical diagnosis of autism, with both positive and negative associations being reported for different sex steroids [8–10].

Prenatally, sex steroid hormone synthesis is regulated by the placenta, which aromatises androgens to estrogens and induces steroidogenesis from the maternal and fetal adrenals [11]. The placenta also affects fetal growth by facilitating nutrient transfer and the production of several growth factors. A “placenta-brain” axis has been proposed, of developmental significance, given that many neurotransmitter precursors (e.g. serotonin) are synthesised in the placenta [12–14].

In cases of clinically diagnosed autism, atypical placental morphology indicating cell excess proliferation [15, 16], increased placental inflammation and increased placental size [17] have been reported in clinical cohort studies. A large epidemiological study of pregnancy complications (*n* = 54,000 autism cases) reported that “placental pathology” was the most likely explanation for the observed association of preeclampsia with low birth weight and autism, as well as with gestational hypertension and autism [18, 19]. A more recent very large epidemiological study (*n* = 23,810 autism cases) replicated this finding and showed that the association of placental syndromes on autism likelihood was, in part, independent to preterm birth and familial likelihood [20].

These studies often do not stratify for sex or report on any sex differences in their epidemiological conclusions. Yet the placenta shows consistent sex differences in terms of both function and susceptibility to dysfunction. Specifically, male placentas show X-linked gene expression differences [21, 22], produce more sex steroid precursors, such as DHEAS at baseline [23], and are more prone to early miscarriage, pregnancy-induced hypertension and spontaneous preterm birth [23–25]. For this reason, sex differences in the placenta have been proposed as a promising area of research [20].

In a previous study of the ‘Generation R’ cohort, we reported significant sex differences in the levels of three placenta-derived markers in maternal serum, as early as the first trimester [26]. Pregnancies of males on average were characterised by significantly lower levels of the placental growth factor (PlGF) and soluble fms-like tyrosine kinase-1 (sFlt-1), even after controlling for placental weight differences between the sexes. PlGF and sFlt-1 have opposing regulating properties on angiogenesis, via activation and suppression of VEGF-related signalling respectively [27, 28]. These markers are produced by the trophoblast, can be measured reliably in the maternal circulation and have been proposed for prenatal screening for a variety of conditions involving placental vascular health, such as gestational hypertension and preeclampsia [29–31]. PlGF levels have also been linked to the sex steroid precursor, DHEAS in experiments in vitro [32]. However, it has not yet been examined if sex differences in these placenta-derived markers are associated with sex differences in neurodevelopmental outcomes and particularly in the number of autistic traits later in children [33]. PlGF has also been proposed to act like a trophic factor in the nervous system, inducing neuronal overgrowth in animal models [27], a phenotype which has also been associated with autism [34–36].

‘Generation R’ is a longitudinal birth cohort of almost 10,000 individuals in Rotterdam, the Netherlands that also monitors the children’s development, from birth to adolescence. Given the novel and significant sex differences in the levels of placental-derived markers that were reported previously, we aimed to examine:

1. If observed sex differences are consistent in the 2^nd^ trimester, following the activation of the male testes.
2. If the levels in these placenta-derived markers in the 1^st^ and 2^nd^ trimester are associated with children’s later number of autistic traits and/or an autism diagnosis.
3. If sex differences in these placenta-derived markers mediate sex differences in children’s autistic traits, in healthy and complicated pregnancies.

We hypothesize that sex differences in placenta-derived markers partially mediate sex differences in neurodevelopment, and that marker concentrations found more commonly in males are also associated with higher autistic traits.

## Methods

### The cohort

This project utilised data that were collected as part of the ‘Generation R Study’, a prospective cohort of expectant women and their children, in Rotterdam, NL. The protocol and details of the study have been reported in detail elsewhere [37]. Participants consented to use their samples and clinical information for the identification of environmental or genetic parameters that contribute to developmental and health-related outcomes. The children of this cohort are followed-up regularly, with both in-person and questionnaire-based measures of their development.

**Fig. 1:**
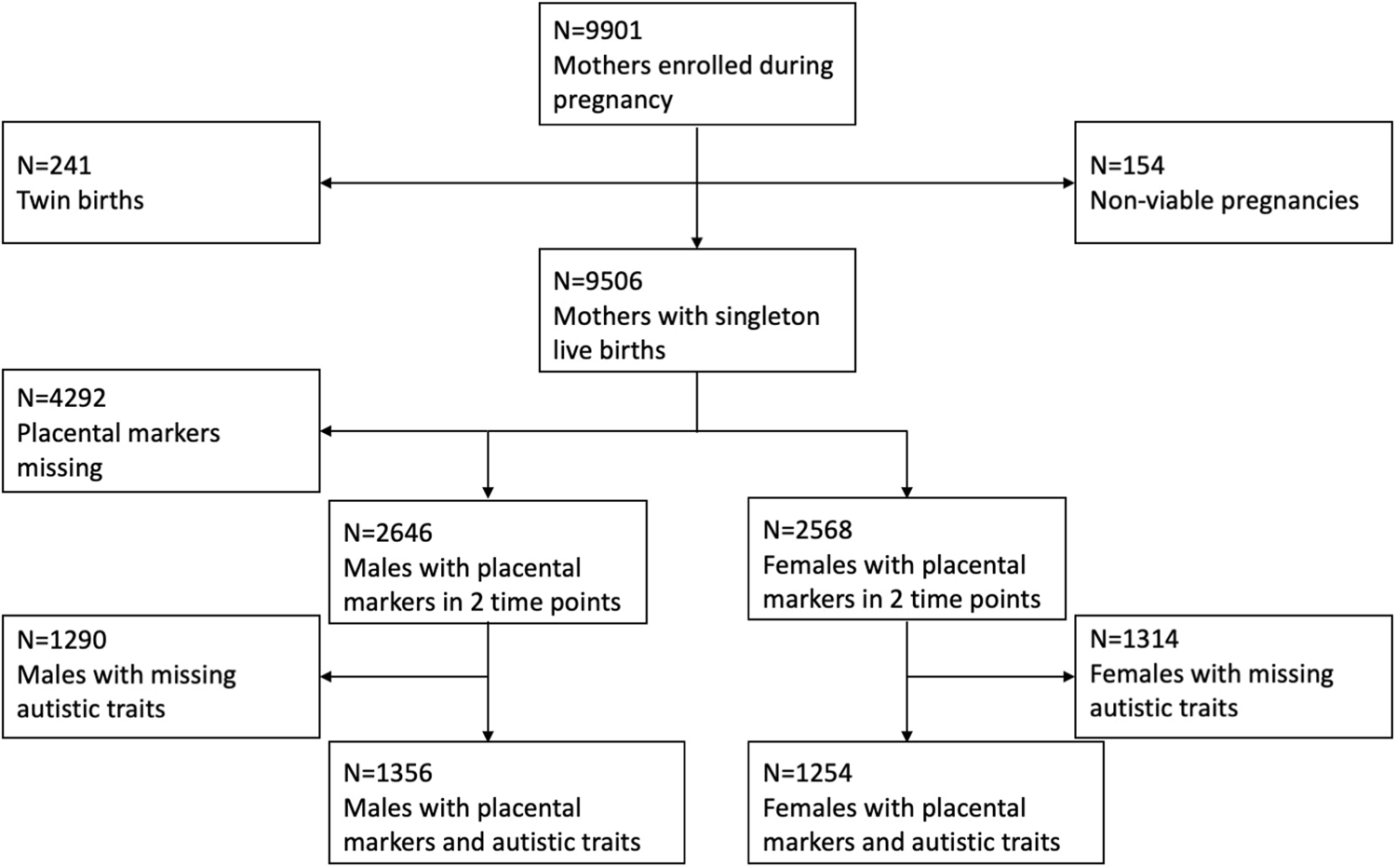
Flowchart of the study. Boxes show sample sizes used for comparison of placenta-derived markers in association with autistic traits and a diagnosis of autism.

For this analysis, only singleton live births were included (Fig. 1). These corresponded to deliveries that took place between April 2002 and January 2006. Other cohort characteristics have been described before [37], with a breakdown for fetal sex included in Supplementary Table 1. With regards to ethnicity, participating mothers and fathers reported parental national origin based on classifications recommended by ‘Statistics Netherland’ and this was further divided into groups for the purposes of statistical analysis [37]. In addition to the initial written, informed consent by the participating mothers, this study was approved by the review board and the Medical Ethics Committee of the Erasmus Medical Centre.

### Placenta-derived markers and clinical information

The concentrations of sFlt-1 and PlGF were measured in maternal plasma derived from venous blood samples, on two separate occasions during pregnancy, at the end of the first (mean=13.5 [SD = 2.0] weeks gestation), and second trimester (mean= 20.6 [SD = 1.2] weeks gestation). Measurements were with immune electrochemoluminence assay on the Architect System (Abbott Diagnostics B.V., Hoofddorp, The Netherlands) in ng/ml and pg/ml, respectively, and were performed using internal controls. Further details on these protocols of recruitment and sample processing have been previously reported [38]. Processing and weighing of placentas after labour was conducted by specialist midwives and has also been described previously [39].

Demographic data on maternal age, ethnicity, educational level and clinical history were obtained through a self-administered questionnaire at recruitment, with a response rate of 93%. Clinical information about the pregnancies, including data on birth weight, gestational age at blood draws and at birth, as well as about birth complications, were obtained from medical records, completed by community midwives and obstetricians. Specifically, preeclampsia (PE) was defined according to international guidelines on blood pressure elevation (140/90 mmHg or greater), in combination with proteinuria after the 20^th^ gestational week. Pregnancy-induced hypertension (‘PIH’) was defined according to the criteria of the International Society for the Study of Hypertension in Pregnancy, namely hypertension (140/90 mmHg or greater measured in clinic) arising de novo after 20 weeks gestation in the absence of proteinuria and without biochemical or haematological abnormalities [40]. Spontaneous preterm birth (’SPB’) was defined as non-induced delivery onset before the completion of 37th week of gestation for known (e.g. concurrent diagnosis of preeclampsia) or unknown reasons. Designation of ‘small for gestational age’ (from here on “SGA”) was defined as a sex and gestational age-adjusted birthweight below the 10th percentile.

### Autistic traits

The score on the Social Responsiveness Scale (from here on “SRS”) is derived from a questionnaire, comprised of items detailing social motivation, interaction, communication, and autism-related behavioural traits that are specific to the population in question [41]. In this study, participating parents were invited to respond to an 18-item abridged version of the questionnaire for children, when their participants’ children were 6 years old. Items were scored on a Likert scale; 0 (not true); 1 (sometimes true); 2 (often true); and 3 (almost always true). The abridged 18-item questionnaire has been previously described in published Generation-R studies, and has correlation of over 0.93 to the full SRS and a Cronbach’s α-value of 0.92 [42, 43]. Higher scores indicate greater challenges with social communication and more autism-related behavioural traits. Additional details on recruitment and neurodevelopmental follow-ups of the children in ‘Generation R’ have been previously reported [37].

### Autism diagnoses

All diagnoses of the children were made in the community by specialised healthcare professionals and according to the Diagnostic and Statistical Manual of Mental Disorders (DSM) IV/5 or the International Classification of Primary Care (ICPC) criteria. Linkage to the children’s ‘Generation R’ records was achieved following targeted contact of the families’ dedicated general practitioners. They are required by the Dutch healthcare regulatory authorities to collect all records and assessments of their patients conducted within the practice or by specialised services, as is the case for autism diagnoses. General practitioners were contacted in order to submit these records in a targeted assessment that prioritised children who had scored high in questionnaires of neurodevelopmental deficits during the study (the CBCL and SCQ - with sex-specific thresholds), as well as children whose parents had reported that the child had undergone a diagnostic assessment for autism, at any point during their participation in the study. This work-up of specialist records to obtain autism diagnoses took place before study participants had reached 9 years of life. The mean age of diagnosis was 6 years of age, as described in previous Generation-R publications [42].

### Statistical analysis

Baseline characteristics were compared between males and females with Mann-Whitney U-tests or Chi-squared tests where appropriate. Multivariate imputations by chained equations (MICE algorithm) were used to impute missing values for demographic variables of the mothers and children. These included maternal age, BMI, educational attainment, and ethnicity, as well as birth weight and the age of SRS measurement for the children.

The concentrations for sFlt-1 and PlGF were compared between the sexes, via pairwise Mann-Whitney U-tests, as well as via multiple linear regression models. To facilitate statistical comparisons with the latter method, distributions of plasma-derived placenta-derived markers were first log-transformed as the dependent variable, with fetal sex, gestational age (at the time of plasma collection) and placental weight (at birth) as independent predictors. Concentrations for sFlt-1 and PlGF were compared between trimesters via Mann-Whitney U-tests. In addition, the change of PlGF concentration between the two time-points (“PlGF-change”) of measurement was computed with the following model:

$$PlGF {\hbox{-}} change = \left( {\left[ {PlGF} \right]\_t2 - \left[ {PlGF} \right]\_t1} \right)/\left( {gestational \, age\_t2 - gestational \, age\_\_t1} \right),$$

with ‘t1’ denoting measurement in the first and ‘t2’ in the second trimester.

With regard to autistic traits, z-scores of SRS scores were computed according to the properties of their distribution in the entire cohort (from here on “autistic traits”). Extreme outliers (*n* = 95) were then reduced to a maximum value specified by adding three times the interquartile range (IQR) to the upper quartile of the IQR (SRS z-score = 3.1).

For the association of placental proteins with autistic traits, every placental variable (including the rate of PlGF change) was untransformed and studied separately in three multivariable regression models. Model 1 controlled for the sex and age of the child at the time of SRS scoring. Model 2 further controlled for the following cohort covariates: maternal age, maternal BMI in the beginning of the pregnancy, maternal ethnicity and maternal education level. Finally, Model 3 was additionally controlled for potential confounder variables that may also be considered mediators; namely total birth weight (adjusted for gestational age at birth by division) and placental weight at birth.

Nominally significant results for Model 3 were further scrutinised in sensitivity analyses, that restricted the cohort to the following categories: First, in pregnancies of European maternal ethnicity, given previously reported differences in SRS scores of potentially cultural origin [44]. Second, in pregnancies without any reported placental or other complications (PIH, PE, SGA, SPB or induced preterm birth). Third, in pregnancies and children without an autism diagnosis by age 6, in order to check if the observed effects were driven chiefly by diagnosed individuals or could be generalised to the undiagnosed population.

In addition, mediation analyses were conducted, which tested for indirect effects of sex on SRS scores, stemming from sex differences in the levels of the placenta-derived markers. In these models, fetal sex was the. predictor, SRS scores were the outcome and concentrations of markers were the mediator. The R package “mediation” was used, set on one thousand simulations and the concentrations of the markers had been previously log-transformed and adjusted for placental weight and gestational age via linear regression.

Finally, in terms of ASD diagnosis (from here on ‘autism’), case-control comparisons of placenta-derived marker levels in maternal serum were conducted in males only, because of the low number of diagnosed females. These were initially compared via Mann-Whitney U-tests, with significant results further analysed via linear regression models, with log-transformed marker concentrations as the dependent variable and the addition of the following independent variables: autism diagnosis, gestational age at measurement, gestational age at birth, placental weight and the presence of pregnancy complications (coded as a binary yes/no variable for PIH, PE, SGA and SPB).

## Results

### Demographic information and neurodevelopmental outcomes

**Fig. 2:**
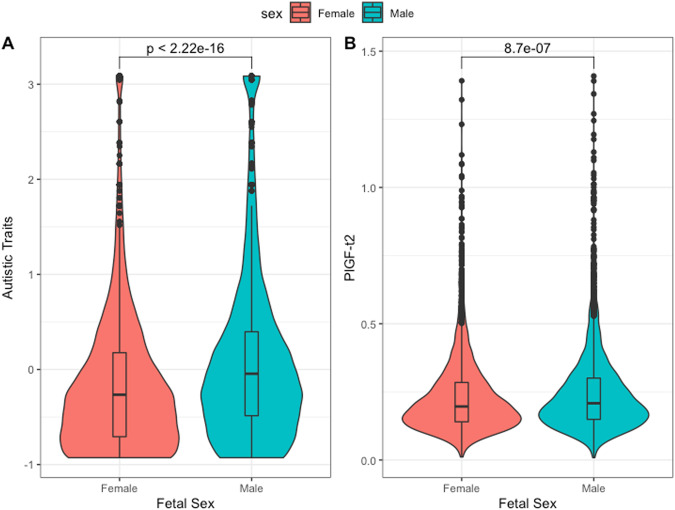
Sex differences in study variables. Males have significantly higher (**A**) autistic traits and (**B**) PlGF levels in maternal plasma at the 2nd trimester. Values are presented in z-scores. P-values are of U-tests of autistic traits and PlGF concentrations, respectively.

The current analysis was restricted to children in the Generation R cohort (*n* = 5214) with, placenta-derived marker measurements in maternal plasma at two time-points of pregnancy in the late first and second trimesters (Fig. 1). In this subset of the cohort, the mean age of the mothers was 31.13 years of age (SD = 4.4) on the first pregnancy appointment, with a mean BMI of 25.3 (SD = 4.0) in the late first trimester and a m/f sex ratio of 1.03:1. A large percentage of the mothers in the cohort was of European ethnicity (80.04%) and of higher education (62.3%) (Supplementary Table 1). Other ethnicities that were represented, in order of frequency, were Turkish (6%), Surinamese (5.6%) and Indonesian (3.4%) [37]. Scores for autistic traits, as measured on the SRS at age 6 (mean age = 74 months, SD = 5.8 months) were available in 3469 children. Autistic traits differed significantly between the sexes, with males scoring significantly higher than females (Cohen’s D = 0.31*, p* < 0.0001)(Fig. 2A). Autistic traits also correlated positively with the age of the child at the time of assessment, and negatively with the age of the mother (Supplementary Table 1).

### Placenta-derived markers in maternal plasma

Levels of both placenta-derived proteins correlated significantly between trimesters (PlGF: Pearson’s *r* = 0.45, *p* < 0.0001; sFlt-1: Pearson’s r = 0.72, *p* < 0.0001) and with each other in varying degrees (2^nd^ trimester PlGF-sFlt-1: Pearson’s *r* = 0.12, *p* < 0.0001)(Supplementary Fig. 1), as well as with a variety of maternal characteristics, including maternal age (for sFlt-1) and maternal BMI at the start of pregnancy (for both) (Supplementary Table 2). Placenta-derived markers showed varying degrees of change between the late first and second trimesters. sFlt-1 increased marginally between the 1^st^ and 2^nd^ trimesters (U-test, *p* = 0.028). On the contrary, PlGF increased more sharply between the two trimesters (U-test *p* < 0.001)(Supplementary Fig. 2).

Sex differences were assessed via pairwise Mann-Whitney U-tests (Table 1) and with multiple regression models that controlled for gestational age and placental weight (Supplementary Table 3). In the 1^st^ trimester, as previously reported, both placenta-derived markers were significantly lower in pregnancies of males [38]. In the 2^nd^ trimester, sFlt-1 levels continued to be significantly lower in the pregnancies of males, compared to females. On the contrary, PlGF levels in the second trimester were significantly higher in males (Fig. 2B).

**Table 1:** Placental proteins in maternal plasma at two time-points of measurement (1st: mean=13.5 weeks, 2nd: mean= 20.6 weeks), and a longitudinal variable corresponding to the rate of PlGF change between them.

1^st^ trimester	N	Mean	Mean Males	Mean Females	*p* value for sex difference
**PlGF ng/ml**	2912	0.054	0.053	0.056	0.025
**sFlt-1 ng/ml**	2910	5.65	5.35	5.98	<0.0001
2^nd^ trimester					
**PlGF ng/ml**	3469	0.231	0.236	0.224	0.0027
**sFlt-1 ng/ml**	3467	5.88	5.59	6.20	<0.0001
Longitudinal					
**PlGF - change**	2627	0.025	0.026	0.024	0.0014
**sFlt-1 change**	2623	0.015	0.014	0.016	0.913

*P* values for sex-difference correspond to pair-wise comparisons via U-tests.

Because of this reversal in the direction of sex differences and the large difference between time-points of measurement, compared to sFLT-1 (Supplementary Fig. 2), the rate of change of PlGF was also modelled as described in the Methods and included in subsequent analyses. This composite measure also showed a significant sex difference, with higher rates in males. By comparison, the change in sFlt-1 between trimesters did not show any sex differences and was not included in further analyses.

### Association of placenta-derived markers with autistic traits

There was no association of sFlt-1 levels at either the first or the second trimester, to the autistic traits of males or females (Table 2). PlGF levels, in the 1^st^ and 2^nd^ trimester, correlated to autistic traits in multiple regression models that controlled for sex and age at point of autistic traits ascertainment (Model 1), as well as cohort covariates (e.g. maternal age, BMI, educational attainment: Model 2). When including potential confounders (birth weight and placental weight: Model 3), this effect was significant in the 2^nd^ trimester (β = 0.244 [SE = 0.112], *p* = 0.03) but not the 1^st^ (Model 3: β = 0.645 [SE = 0.035], *p* *=* 0.068).

**Table 2:** Association of z-scores of child SRS Scores to placental protein concentrations in maternal plasma.

	Association with Autistic Traits (z-score of SRS)											
	MALES				FEMALES				BOTH			
1^st^ trimester	N	β	SE	p	N	β	SE	p	N	β	SE	p
**PIGF**												
Model 1	1504	0.898	0.525	0.087	1408	**1.732**	**0.357**	**<0.0001**	2912	**1.38**	**0.31**	**<0.0001**
Model 2		0.478	0.511	0.351		**1.081**	**0.352**	**0.0022**		**0.795**	**0.302**	**0.008**
Model 3		0.434	0.610	0.477		**0.882**	**0.408**	**0.031**		0.645	0.035	0.068
**sFlt-1**												
Model 1	1502	0.005	0.008	0.525	1408	0.006	0.005	0.213	2910	0.006	0.004	0.198
Model 2		0.006	0.007	0.377		0.004	0.005	0.481		0.005	0.004	0.284
Model 3		0.006	0.009	0.479		0.007	0.006	0.288		0.006	0.005	0.258
2^nd^ trimester												
**PIGF**												
Model 1	1804	**0.378**	**0.137**	**0.006**	1665	**0.386**	**0.117**	**0.001**	3469	**0.384**	**0.090**	**<0.0001**
Model 2		0.181	0.134	0.177		**0.235**	**0.115**	**0.041**		**0.210**	**0.088**	**0.017**
Model 3		0.10	0.18	0.570		**0.393**	**0.143**	**0.006**		**0.244**	**0.112**	**0.030**
**sFlt-1**												
Model 1	1802	0.004	0.005	0.469	1665	−0.001	0.004	0.751	3467	0.001	0.003	0.738
Model 2		0.000	0.005	0.99		−0.003	0.004	0.407		−0.0014	0.003	0.629
Model 3		0.001	0.006	0.836		−0.002	0.005	0.731		−0.0001	0.004	0.984
Longitudinal												
**PIGF-change**												
Model 1	1367	**3.028**	**1.241**	**0.015**	1260	**2.981**	**0.971**	**0.0022**	2627	**3.000**	**0.786**	**0.0001**
Model 2		**2.374**	**1.210**	**0.049**		**1.961**	**0.947**	**0.0387**		**2.067**	**0.765**	**0.007**
Model 3		1.525	1.485	0.305		**3.050**	**1.107**	**0.006**		**2.187**	**0.912**	**0.017**

Model 1 covariates: age of child at SRS measurement. Model 2 covariates: age of child at SRS measurement, maternal age, maternal BMI in the beginning of the pregnancy, maternal ethnicity and maternal education level. Model 3 covariates: as in Model 2 with the addition of potential confounders; placental weight and birth weight-adjusted for gestational age at birth.

Bold values indicates statistical significant *P* values.

In sex-stratified analysis, higher PIGF levels were associated with more autistic traits in females in both the 1^st^ and 2^nd^ trimesters (1^st^ trimester - Model 3: β = 0.882 [SE = 0.408], *p* = 0.031/ 2^nd^ trimester - Model 3: β = 0.393 [SE = 0.143], *p* = 0.006). This was not significant in male-only comparisons when controlling for cohort covariates and confounders (Models 2 and 3). The rate of increase in PlGF levels was positively correlated to the autistic traits in both males and females (Model 3: β = 2.19 [SE = 0.91], *p* = 0.017). In sex-stratified analysis, this was more evident in females, with males showing a significant association in Models 1 and 2 (β = 2.37 [SE = 1.21]*, p* = 0.049), but not in Model 3, which controlled for the effects of birth weight and placental weight (β = 1.52 [SE = 1.49], *p* = 0.305).

Sensitivity analyses were performed by excluding individuals with an autism diagnosis or pregnancies with complications, as these cases could be driving the association between autistic traits and placental markers respectively. In addition, given different ethnicities were represented in the cohort (Supplementary Table 1), an additional analysis was conducted in a subset that was more homogeneous and only included individuals with a European maternal ancestry. These sensitivity analyses had consistent results and showed that the positive association with autistic traits persisted for both PlGF levels in the 2^nd^ trimester and the rate of PlGF increase (Supplementary Table 4).

### Pregnancy complications

**Fig. 3:**
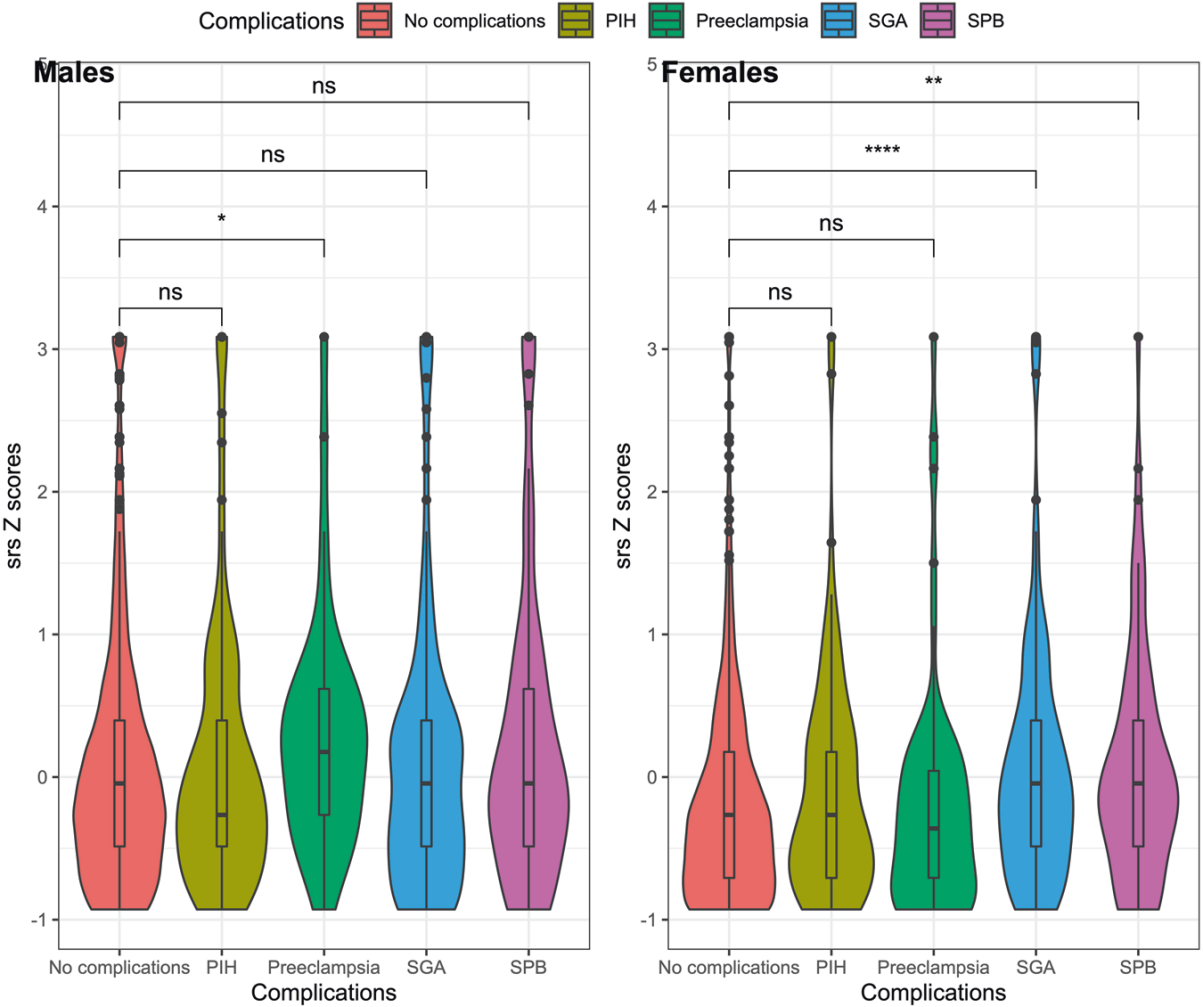
Sex-stratified z-scores of children’s SRS at age 6, corresponding to uncomplicated pregnancies and compared to specific, placenta-related complications via Mann Whitney U-tests. PIH pregnancy-induced hypertension, SGA small for gestational age, SPB spontaneous preterm birth, ns not significant, *p < 0.05, **p < 0.01, ****p < 0.0001.

SRS scores were significantly higher in pregnancies with complications, which may be linked to the placenta (Supplementary Table 5). Male and females showed different patterns driving this susceptibility (Fig. 3). In pregnancies of females, this was driven by individuals born SGA (Cohen’s D = 0.30, U-test *- p* < 0.0001) or after a spontaneous and preterm birth (Cohen’s D = 0.29, U-test-*p* = 0.004). Preeclampsia was a specific risk factor to males (Cohen’s D = 0.23, U-test, *p* = 0.022). These differences corresponded to similar profiles in the levels of placenta-derived markers (Supplementary Table 5), Male pregnancies with PE and females born SGA, both had significantly lower PlGF in the second trimester and a lower rate of PlGF change between trimesters, as well as higher SRS scores, compared to uncomplicated pregnancies.

### Mediation analysis

Mediation analysis (Supplementary Fig. 3) showed that the sex difference in autistic traits (higher SRS in males, Cohen’s D = 0.31, *p* < 0.0001) was significantly mediated by PlGF levels in the 2^nd^ trimester, with higher PlGF levels being linked to higher autistic traits in males more than females (ACME: 0.0005, *p* = 0.004) (Table 3). The rate of PlGF-elevation also mediated part of the association of sex with SRS Scores (ACME: 0.004, *p* = 0.026). This was not found for sFLt-1 at either trimester, despite pronounced sex differences in its concentrations.

**Table 3:** Sex differences in 2^nd^ trimester prenatal PlGF levels mediate a part of the sex differences in SRS scores at age 6.

Placenta-derived markers	*Mediation of the effect of sex on autistic traits*			
1^st^ trimester	ACME (CI)	*p*-value of mediation	ADE (CI)	*p*-value of added effect
**PlGF**	−0.004 (−0.01 - 0.00)	0.10	0.26 (0.21 - 0.31)	<0.0001
**sFlt-1**	−0.003 (−0.01 - 0.00)	0.24	0.26 (0.20 - 0.31)	<0.0001
2^nd^ trimester				
**PlGF**	**0.005 (0.001 - 0.01)**	**0.004**	0.24 (0.19 - 0.29)	<0.0001
**sFlt-1**	−0.001 (−0.01 - 0.00)	0.86	0.25 (0.19 - 0.29)	<0.0001
Longitudinal				
**PlGF - change**	**0.005 (0.00 - 0.01)**	**0.026**	0.24 (0.18 - 0.30)	<0.0001

Bold values indicates statistical significant *P* values.

### Cases of autism

By the time this study was conducted, a total of *n* = 87 (*n* = 12 females) had been diagnosed as autistic in the Generation R cohort. These children had higher autistic traits on the SRS (Cohen’s D = 2.4, U-test - *p* < 0.0001) and were more likely to have experienced pregnancy-induced hypertension (*n* = 8) (χ^2^ = 8.1, *p* = 0.005) but showed no other significant differences in the rates of other pregnancy complications. Of these diagnosed cases, *n* = 64 had available measurements of placenta-derived markers in maternal serum. Case-control comparisons were only conducted in males, as the number of females with a diagnosis and placenta-derived markers was too low (*n* = 9) for a sufficiently powered sex-stratified comparison of placenta-derived marker levels. Compared to undiagnosed males, autistic males had lower s-Flt1 levels in maternal serum at the second trimester (Cohen’s D = 0.25, U-test - *p* = 0.027) (Supplementary Table 6, Supplementary Fig. 4) but no significant differences in PlGF levels or the rate of PlGF change between trimesters. This was further shown in a multiple linear regression model, with log-transformed sFlt-1 levels as the dependent variable, which also controlled for gestational age at time of marker measurement, placental weight, age at birth and the presence of any pregnancy complications (Supplementary Table 7).

## Discussion

This study investigates sex differences in placenta-derived markers of angiogenesis and their association with autistic traits in children. The analysis focused on the levels of the placenta growth factor (PlGF) and the soluble fms-like tyrosine kinase-1 (sFlt-1), which are produced by the placenta and have opposing functions on angiogenesis, via activation or inhibition of the VEGF pathway respectively.

These two placenta-derived markers were previously studied in the late 1^st^ trimester and found to be significantly lower in the pregnancies of males [38]. In this study, we find sex differences in their concentrations continue to be significant in the 2^nd^ trimester, independently of placental weight differences. Specifically, sFlt-1 continues to be significantly lower in males, with little change between time-points of measurement. However, the levels of the placental growth factor (PlGF), are significantly higher in males, due to faster increase from the late first to the second trimester.

In addition, we show, for the first time, that sex differences in PlGF levels are linked to sex differences in autistic traits in the general population. Specifically, we find that high levels of PlGF are associated with higher autistic traits when controlling for sex. In sex-stratified analyses, this association was statistically significant for females but not for males. In addition, it was found that higher PlGF may also mediate higher autistic traits in males more than in females.

The steeper increase of PlGF into the 2^nd^ trimester in males could be attributed to the effects of fetal androgens, which become rapidly elevated in males during mid-pregnancy, following the activation of the testes [2]. In cellular and human studies outside of pregnancy, PlGF levels have been found to correlate to the levels of steroid derivatives and DHEAS [32, 45]. In turn, DHEAS is also significantly higher in the placentas of males, as shown via RNA-Seq in a large clinical cohort [23].

It is as yet unclear how higher levels of PlGF prenatally may affect neurodevelopment and lead to higher autistic traits in the children. The observed association may be due to an underlying factor, such as sex steroid hormones, which may be affecting both PlGF levels [32] and neurodevelopment [46, 47]. Alternatively, PlGF could potentially affect the developing brain directly, by increasing the proliferation of Schwann cells or by interacting with the maturation of the blood-brain-barrier, as shown in previous studies [27, 48]. Consistent with this, high PlGF concentrations correlate with higher infant growth rates of body weight and head circumference in this and other cohorts [26, 49]. Higher levels of PlGF could thus be interpreted as part of a normative or adaptive process. This would be consistent with the angiogenic and neuroproliferative effects of the factor, as well as in vitro evidence that PlGF is upregulated following induced hypoxia [27].

Placental dysfunction, such as in the case of preeclampsia, has been previously associated with lower, rather than higher, levels of PlGF [31] and the same was found in this cohort (Supplementary Table 5). Sex differences in PlGF levels are also absent in complicated pregnancies [26]. Therefore, the process leading to an increase in PlGF levels in the 2^nd^ trimester, as well as to higher PlGF levels in males, appears to be absent in cases of placental dysfunction. This may be the reason why, in this study, the positive association of PlGF to autistic traits, had a higher effect size when complicated pregnancies were excluded from the cohort (Supplementary Table 4). This finding could also be consistent with an adaptive role for high levels of PlGF in neurodevelopment. Additional research is required in order to explore these speculations.

In addition, a comparison of placental marker concentrations was conducted between cases and controls for a diagnosis of autism. No significant differences were found in PlGF levels. However, the levels of sFlt-1 were significantly lower in autistic males compared to undiagnosed males, who in turn had significantly lower sFlt-1 levels to undiagnosed females. In-vitro experiments have also shown that sFlt-1 is downregulated by hypoxia in cultured endothelial cells [28]. Given the small sample size of this case-control comparison, additional studies are needed in an independent cohort as well as in autistic females, in order to increase confidence in this finding.

Cases of autism in this cohort have higher autistic traits but they may also be characterised by higher rates of learning difficulties, leading to referrals and a diagnosis in early childhood. Undiagnosed individuals with high autistic traits may still receive a diagnosis in later life, but may also have developed adaptive behaviours (e.g. camouflaging), which may be absent in diagnosed autistic children [50, 51]. Therefore the findings regarding autistic traits may not always be specific to autism [52] or correspond to significant differences in case-control comparisons [42].

This study is further limited by potential ascertainment bias, as the population was restricted to pregnant women who consented not only to prenatal testing but also to long-term follow-ups of their children [53]. In addition, the mothers of the Generation R cohort are largely urban and of a relatively high socioeconomic status. While these aspects have been discussed before [37] and sensitivity analyses were conducted in this study, it is important to note that the results may not generalise to other populations. Furthermore, the reported effect sizes for PlGF are small, particularly for the observed mediation on sex differences, indicating that other regulatory molecules may be more informative for autistic traits. Finally, the case-control comparison for autism had limited power and was only possible in males. The longitudinal nature of the cohort may also mean that the two groups overlap and that individuals without a diagnosis may receive one later in life. Further research is needed in order to replicate these findings in independent cohorts, in other type of samples (e.g. amniotic fluid), in autistic females and to study the interaction of placenta-derived markers with steroid hormones and genetic factors.

In conclusion, this study has shown that the upregulation of the placenta growth factor differs between the sexes and is associated with higher autistic traits in childhood. This phenomenon may offer insight into how the placenta regulates neurodevelopment in males and females.

## Supplementary information

Supplementary Material

## Data Availability

The datasets generated during and/or analysed during the current study are not publicly available due to limited ethics approval for the wider clinical study (‘Generation R’) and due to the specific consent provided by the participants. They may be available from the corresponding author on reasonable request and pending approval of any future analyses.
