# Supplementary material for: Sex differences in placenta-derived markers and later autistic traits in children
Source: Transl Psychiatry. 2023 Jul 13;13:256. doi: 10.1038/s41398-023-02552-w (PMC10344956; doi:10.1038/s41398-023-02552-w)
Supplement: Supplementary file 1 — Supplementary Material [file 41398_2023_2552_MOESM1_ESM.pdf]

Supplementary Material

Supplementary Figure 1: *Heatmap of the pairwise correlations of all assayed placental markers in maternal plasma. All values are Pearson's correlation coefficients, significant at p<0.001. t1: 1st trimester measurement / t2: 2nd trimester measurement*

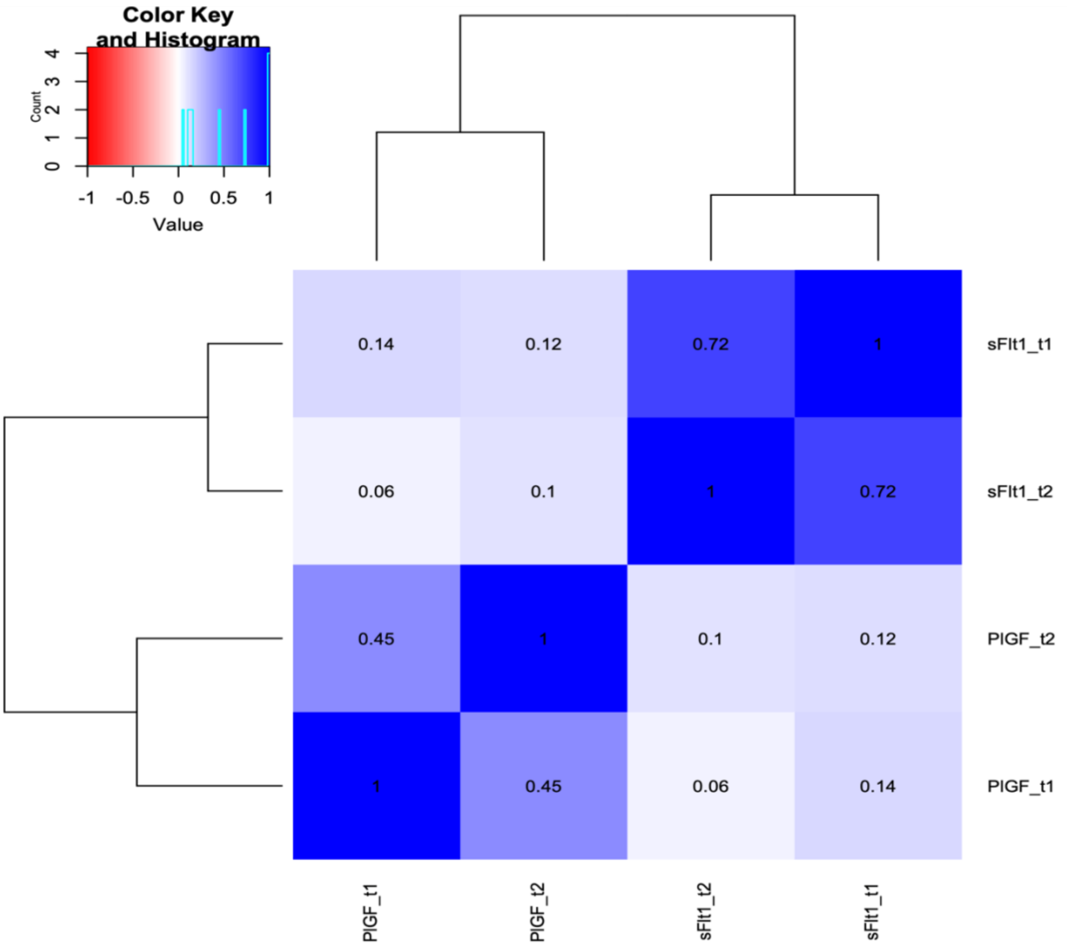

Supplementary Table 1*: Maternal characteristics in the study cohort (a subset of Generation R), their differences according to fetal sex (p-value for Mann Whitney U-test) and their association to z-scores of SRS Scores (values for univariate linear regression models). *BMI at the start of pregnancy.*

**Cohort n= 5,214**	*Age of child (months)*	*Maternal age (years)*	*Maternal BMI**	*Maternal Education*	*Birth Weight (gr)*	*Placental Weight (gr)*
Missing %	0%	0%	15.2%	4%	0.1%	36%
	mean=73.51 SD=5.4	mean=31.13 SD=4.43	mean=25.29 SD=3.98	1: Primary or less, n=75<br>2: Secondary, n=1041<br>3: Higher, n=1889	mean=3423.9 SD=565.96	mean=635.75 SD=146.79
Males n= 2646	mean=73.53 SD=5.45	mean= 31.18 SD=4.48	mean=25.36 SD=4.06	1: n=33<br>2: n=516<br>3: n=961	mean= 3482.9 SD=574.5	mean=642.02 SD=145.0
Females n= 2568	mean=73.50 SD=5.43	mean= 31.08 SD=4.38	mean=25.22 SD=3.89	1: n=42<br>2: n=525<br>3: n=928	mean= 3363.4 SD= 550.63	mean=628.17 SD=150.1
Sex Difference	p=0.651	p=0.420	p=0.904	p=0.534 (for higher)	**p<0.0001**	**p=0.012**
SRS Scores						
β to SRS	**0.10**	**-0.127**	**0.076**	**-0.248**	**-0.058**	-0.0327
p-value	**<0.0001**	**<0.0001**	**<0.0001**	**<0.0001**	**<0.0001**	0.06

Supplementary Table 2: *Maternal characteristics and their correlation to placental marker concentrations in the 1st and 2nd trimester (via Pearson's) . *BMI at the start of pregnancy.*

	*Gestational age**	*Maternal age*	*Maternal BMI**	*Placental Weight*	*Birth Weight*
1st trimester					
PlGF					
β	**0.713**	0.019	0.007	**0.059**	0.008
p-value	**<0.0001**	0.277	0.60	**0.0001**	0.644
Sflt-1					
β	**0.071**	-0.027	**-0.142**	**0.130**	**0.084**
p-value	**<0.0001**	0.123	**<0.0001**	**<0.0001**	**<0.0001**
2nd trimester					
PlGF					
β	**0.315**	-0.01	**-0.119**	**0.072**	**0.047**
p-value	**<0.0001**	0.541	**<0.0001**	**<0.0001**	**0.003**
Sflt-1					
β	**0.024**	**-0.084**	**-0.154**	**0.068**	0.018
p-value	**0.044**	**<0.0001**	**<0.0001**	**<0.0001**	0.264
Longitudinal					
PlGF - change					
β	NA	-0.008	**-0.10**	**0.047**	-0.004
p-value		0.645	**<0.0001**	**0.004**	0.835
sFlt-1 - change					
β	NA	**-0.080**	**-0.058**	**-0.064**	**-0.082**
p-value		**<0.0001**	**<0.0001**	**<0.0001**	**<0.0001**

Supplementary Table 3*: Full linear Regression models for sex differences of placental marker concentrations, controlling for placental weight and gestational age. Marker levels have been log-transformed as the dependent variable.*

	N	*Sex*	*Placental Weight*	*Gestational Age*	*MR Model*
1st trimester					
PlGF	5963	β=-0.0461	β=0.0002	β=0.2389	Adj.R^2= 0.5174
		p=0.0011	p < 0.0001	p < 0.0001	p= <0.0001
sFlt-1	5951	β=-0.0859	β=0.0005	β=0.0227	Adj.R^2=0.0289
		p<0.0001	p<0.0001	p<0.0001	p<0.0001
2nd trimester					
PlGF	7294	β=0.055	β=0.0002	β=0.1408	Adj.R^2=0.098
		p=0.0001	p<0.0001	p<0.0001	p<0.0001
sFlt-1	7292	β=-0.0776	β=0.0003	β=0.0084	Adj.R^2=0.0081
		p<0.0001	p<0.0001	p=0.256	p<0.0001
Longitudinal					
PlGF - change	5250	β=1.6421	β=0.0058	N/A	Adj.R^2=0.0036
		p=0.0072	p=0.0051		p=0.0004
sFlt-1 - change	5236	β=0.0043	β=-0.00018	N/A	Ad.R2=0.0036
		p=0.743	p=<0.0001		p=0.0004

Supplementary Figure 2*: Change of placenta-derived markers for (A) sFlt-1and (B) PlGF, for male and female pregnancies, as indicated by two time-points of measurement at the late 1st and 2nd trimester.*

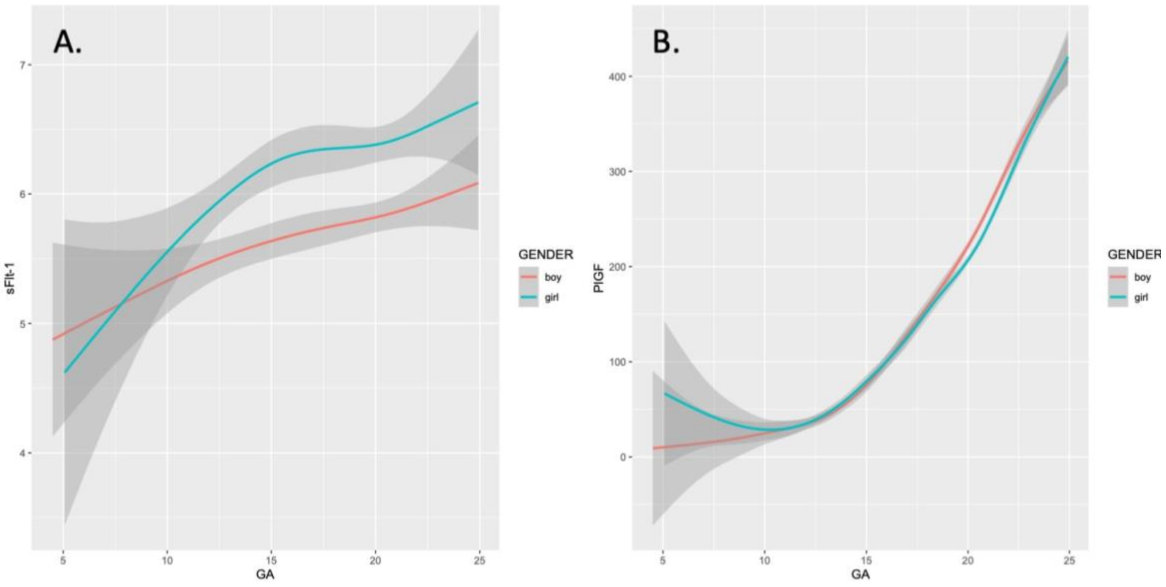

Supplementary Figure 3: *Mediation of placenta-derived markers on sex differences in autistic traits ACME: average causal mediated effect, ADE: average direct effect , CI: confidence interval*

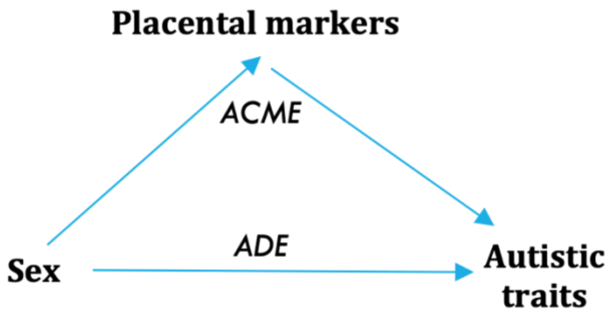

Supplementary Table 4*: Sensitivity Analyses for PlGF at the 2nd trimester, in association with autistic traits (SRS z-scores). Model 1 covariates: age of child at SRS measurement. Model 2 covariates: age of child at SRS measurement, maternal age, maternal BMI in the beginning of the pregnancy, maternal ethnicity, birth weight adjusted for gestational age and maternal education level. Model 3 covariates as in Model 2 and in addition: placental weight at birth, and total birth weight-adjusted for gestational age at birth.*
**European ethnicities include the following categories: Dutch, American-western, Asian-western, Turkish, European, Oceanian.*
***Complications excluded include: Pregnancy-induced Hypertension, Preeclampsia, Born small for gestational age and Spontaneous Preterm Birth.*

PlGF - 2nd trimester to SRS	*FEMALES*			*MALES*			*BOTH*		
	N	β (SE)	p	N	β	p	N	β	p
European ethnicities									
Model 1	1224	**0.279 (0.13)**	**0.045**	1317	0.137 (0.17)	0.429	2541	**0.212** (0.11)	**0.050**
Model 2		**0.270 (0.14)**	**0.049**		0.085 (0.17)	0.619		0.187 (0.10)	0.086
Model 3		**0.367 (0.16)**	**0.025**		0.095 (0.21)	0.652		0.237 (0.13)	0.073
No autism									
Model 1	1657	**0.403 (0.12)**	**0.0008**	1760	**0.403 (0.13)**	**0.003**	3417	**0.206 (0.02)**	**<0.0001**
Model 2		0.221 (0.12)	0.061		0.184 (0.13)	0.157		**0.204 (0.08)**	**0.020**
Model 3		**0.292 (0.14)**	**0.037**		0.178 (0.16)	0.266		**0.227 (0.11)**	**0.032**
No complications									
Model 1	1233	**0.528 (0.14)**	**0.0001**	1179	**0.379** (0.18)	**0.0006**	2412	**0.451 (0.11)**	**<0.0001**
Model 2		**0.360 (0.14)**	**0.0089**		0.151 (0.18)	0.412		**0.249 (0.12)**	**0.031**
Model 3		**0.555 (0.17)**	**0.0015**		-0.049 (0.24)	0.838		0.262 (0.15)	0.076

Supplementary Table 5: *Sex-stratified comparison of placental-derived markers and autistic traits, in maternal plasma between uncomplicated pregnancies and cases with specific complications. P-values correspond to Mann Whitney U-tests comparing to the uncomplicated pregnancies.*

FEMALES	*Mean UC n=4185*	*Mean PIH n=157*	*Mean PE n=108*	*Mean SGA n=269*	*Mean SPB n=195*
1st trimester					
PlGF pg/ml	60.31	**51.67**	**51.79**	55.70	69.40
		**p=0.002**	**p=0.019**	p=0.252	p=0.277
s-Flt1 ng/ml	6.147	5.907	5.703	**5.762**	6.05
		p=0.542	p=0.143	**p=0.012**	p=0.287
2nd trimester					
PlGF pg/ml	235.96	**207.39**	**178.99**	**224.73**	248.43
		**p=0.017**	**p<0.001**	**p=0.01**	p=0.464
s-Flt1 ng/ml	6.44	5.60	6.78	6.94	6.82
		p=0.086	p=0.499	p=0.576	p=0.617
Longitudinal					
PlGF - change	25.30	22.06	**19.80**	**25.01**	26.66
		p=0.133	**p<0.001**	**p=0.04**	p=0.605
Postnatal					
SRS Scores	-0.186	-0.104	-0.210	**0.035**	**0.024**
		p=0.303	p=0.45	**p=<0.0001**	**p=0.004**
MALES	*n=4319*	*n=164*	*n=86*	*n=259*	*n=224*
1st trimester					
PlGF pg/ml	60.08	**44.43**	**53.99**	**53.04**	**53.05**
		**p<0.001**	**p=0.002**	**p=0.040**	**p=0.008**
s-Flt1 ng/ml	5.68	**5.04**	5.51	5.07	5.25
		**p=0.004**	p=0.999	p=0.017	p=0.19
2nd trimester					
PlGF pg/ml	250.64	**208.49**	**212.09**	248.74	267.88
		**p<0.001**	**p=0.010**	p=0.251	p=0.454
s-Flt1 ng/ml	5.90	**4.91**	**7.60**	5.30	6.62
		**p<0.001**	**p=0.018**	p=0.077	p=0.264
Longitudinal					
PlGF - change	27.28	**23.34**	**21.18**	27.06	29.76
		**p=0.003**	**p<0.001**	p=0.582	p=0.658
Postnatal					
SRS Scores	0.085	0.178	**0.291**	0.197	0.211
		p=0.894	**p=0.022**	p=0.136	p=0.252

Supplementary Table 6: *Comparison of placental protein concentrations in maternal plasma between males diagnosed with autism (n=56) and undiagnosed males with available placenta-derived markers in maternal serum (n=2590). P-values correspond to Mann Whitney U-tests.*

	Mean males no-diagnosis	*Mean Autism (n= 56 males)*	*Cohen's D*	*p-value*
1st trimester				
PlGF pg/ml	59.05	54.63	0.089	0.514
s-Flt1 ng/ml	5.62	4.90	0.231	0.146
2nd trimester				
PlGF pg/ml	249.53	226.42	0.150	0.483
s-Flt1 ng/ml	5.89	4.83	0.245	**0.027**
Longitudinal				
PlGF - change	27.11	25.52	0.082	0.904
Postnatal				
SRS Scores	0.06	1.86	2.083	**p<0.0001**

Supplementary Figure 4*: Values of s-Flt1 (log-transformed). Males diagnosed with autism have significantly lower levels of s-Flt1 in maternal serum in the 2nd trimester (Mann Whitney U-test: p=0.027).*

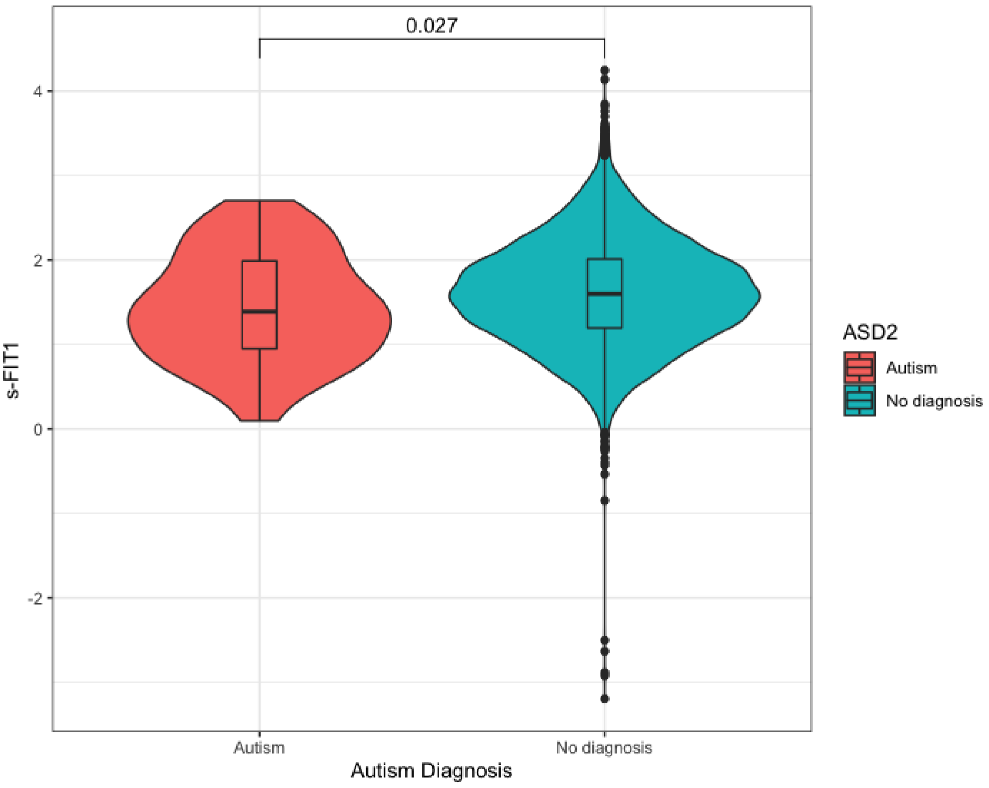

Supplementary Table 7: *Full linear regression model of sFlt-1 levels in the 2nd trimester, in association with a diagnosis of autism in males (n=59). Sflt-1 levels have been log-transformed as the dependent variable.*

Log sFlt-1				
	Coefficient	SE	z-value	p-value
Intercept	β=2.078	0.349	5.96	**<0.001**
Autism	β=0.002	0.091	-2.15	**0.032**
GA at measurement	β=-0.196	0.010	0.21	0.837
GA at birth	β=-0.018	0.007	-2.53	**0.011**
Placental weight	β=0.000	0.000	3.46	**0.001**
Complications (yes/no)	β=-0.073	0.037	-1.97	**0.049**
	F-statistic: 4.687 on 5 and 2679 df, **p=0.0003**			
